# Museomics Clarifies the Classification of *Aloidendron* (Asphodelaceae), the Iconic African Tree Aloes

**DOI:** 10.3389/fpls.2019.01227

**Published:** 2019-10-15

**Authors:** Panagiota Malakasi, Sidonie Bellot, Richard Dee, Olwen M. Grace

**Affiliations:** Comparative Plant & Fungal Biology, Royal Botanic Gardens, Kew, Surrey, United Kingdom

**Keywords:** aloe, botanic garden, evolution, herbarium, phylogenomic analysis, systematics

## Abstract

Arborescent succulent plants are regarded as keystone and indicator species in desert ecosystems due to their large stature and long lifespans. Tree aloes, the genus *Aloidendron*, are icons of the southern African deserts yet have proved elusive subjects due to the difficulty of obtaining material of known provenance for comparative study. Consequently, evolutionary relationships among representatives of the unusual arborescent life form have remained unclear until now. We used a museomics approach to overcome this challenge. Chloroplast genomes of six *Aloidendron* species and 12 other members of Asphodelaceae were sequenced from modern living collections and herbarium specimens, including the type specimens of all but two *Aloidendron* species, the earliest of which was collected 130 years ago. Maximum-likelihood trees estimated from full chloroplast genomes and the nuclear internal transcribed spacer (ITS) region show that *Aloidendron sabaeum*, from the Arabian Peninsula, is nested within *Aloe* while the Madagascar endemic *Aloestrela suzannae* is most closely related to the Somalian *Aloidendron eminens*. We observed phylogenetic conflicts between the plastid and nuclear topologies, which may be indicative of recurrent hybridisation or incomplete lineage sorting events in *Aloe* and in *Aloidendron*. Comparing species ecology in the context provided by our phylogeny suggests that habitat preference to either xeric deserts or humid forests/thickets evolved repeatedly in *Aloidendron*. Our findings demonstrate the value of botanical collections for the study and classification of taxonomically challenging succulent plants.

## Introduction

**Figure 1:**
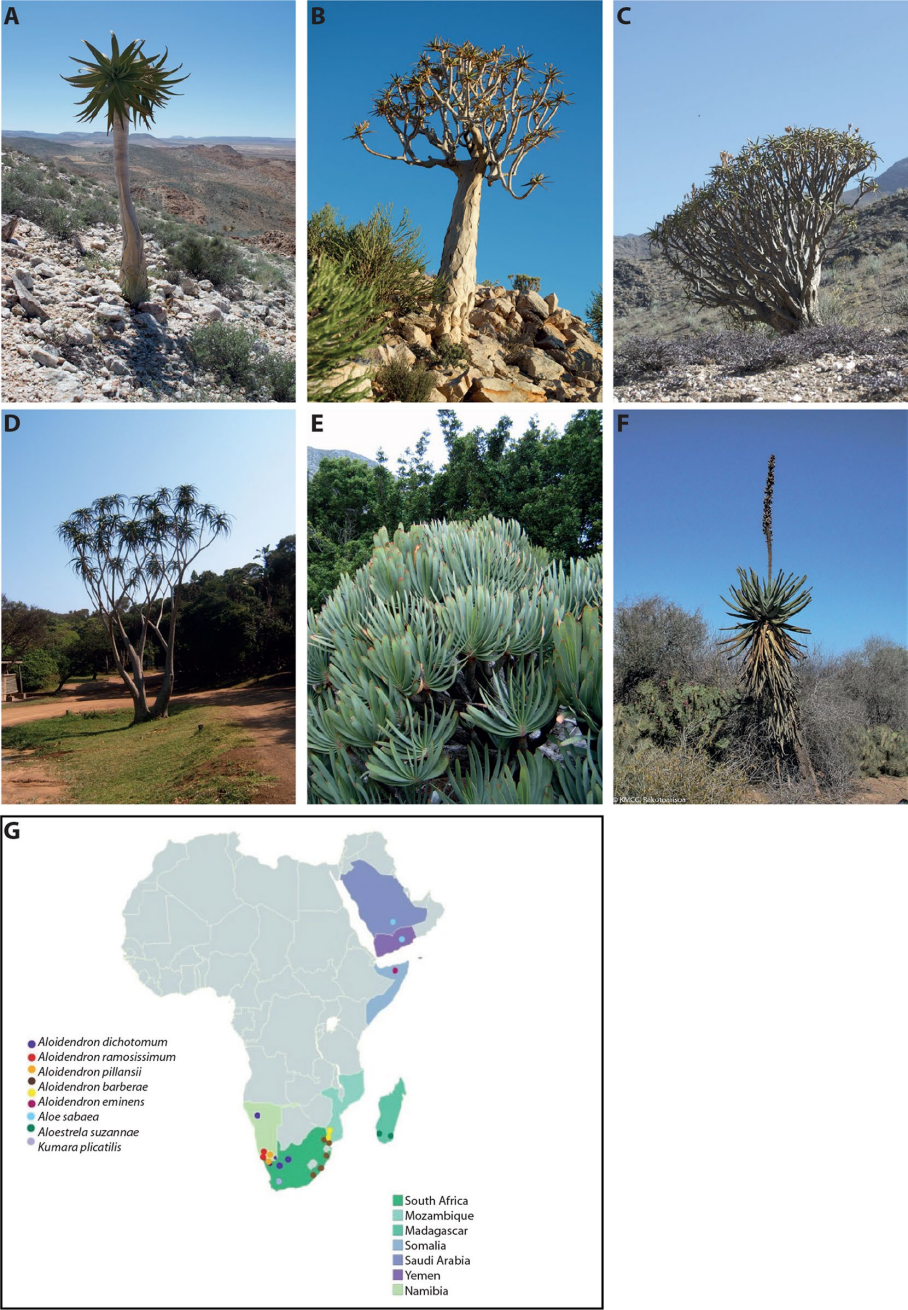
The genus *Aloidendron* comprises seven species characterised by an arborescent habit and succulent leaves (photographs O.M. Grace unless otherwise stated). Three iconic species are endemic to the southern African deserts **(A)** *Aloidendron pillansii*, **(B)** *Aloidendron dichotomum* and **(C)** *Aloidendron ramosissimum*, while **(D)** *Aloidendron barberae* in KwaZulu-Natal is one of three species found in humid coastal thicket and forest in southern Africa, along with *Aloidendron eminens* (not shown) in Somalia and the Arabian Peninsula. Two iconic taxa sometimes regarded as tree aloes include **(E)** *Kumara plicatilis*, a distinctive Cape endemic and **(F)** *Aloestrela suzannae*, a distinctive unbranched arborescent species endemic to the spiny forest of Madagascar (photograph R.S. Rakotoarisoa). **(G)** Distribution map of the seven species of tree aloes, *A. suzannae* and *K. plicatilis*.

The presence of water-storing tissues in plants is a common adaptation to drought that has evolved multiple times. The condition, known as succulence, is most commonly associated with two categories in Raunkier's (Raunkier, 1934) classification of plant life forms: nanophanerophytes (having woody stems, persisting for several years, and having buds below 3 m) and diminutive chamerophytes (having herbaceous or woody stems, persisting for several years, and having buds on or near soil level and not above 50 cm). Relatively few succulent plants are arborescent phanerophytes (having woody stems, persisting for several years, having buds above 3 m) (Raunkier, 1934), and tree-like succulents are therefore conspicuous in the arid landscape. They are sometimes regarded as keystone and indicator species for the habitats in which they occur due to their large stature and long lifespans. Examples include the saguaro cactus *[Carnegiea gigantea* (Engelm.) Britton & Rose, Cactaceae] in the Sonoran desert, the candelabra tree [*Euphorbia ingens* E. Mey. (Euphorbiaceae)] in the subtropical drylands of southern Africa (Van Der Linde et al., 2012), and the quiver tree [*Aloidendron dichotomum* (A. Berger) Klopper & Gideon F.Sm. (Asphodelaceae subf. Alooideae)]. The latter, an icon of the Namib desert, has been proposed as an indicator for species migration associated with anthropogenic climate change (Foden et al., 2007; Jack et al., 2016). Here, we focus on the genus *Aloidendron*. *Aloidendron* comprises seven species known colloquially as tree aloes, due to their distinctive arborescent habit with dichotomous branching and compressed terminal rosettes of succulent leaves (**Figure 1**), and its close relationship to the (∼500 species) of the leaf-succulent genus *Aloe*. Two other iconic arborescent aloes are sometimes included in a broad concept of tree aloes: the dichotomously branched Cape endemic *Kumara plicatilis* (L.) G.D. Rowley and the (usually) unbranched species *Aloestrela suzannae* (Decary) Gideon F.Sm., which is restricted to the arid spiny thicket of southern Madagascar.

All species of *Aloidendron* are confined to Namibia, South Africa and Mozambique, with two exceptions: *Aloidendron eminens* (Reynolds & P.R.O. Bally) Klopper & Gideon F.Sm., endemic to Somalia, and *Aloidendron sabaeum* (Schweinf.) Boatwr. & J.C. Manning, only found in the Arabian Peninsula (**Figure 1**). Until now, it has not been clear whether the disjunct distribution of *Aloidendron* in the southern and northern native range limits of the closely related genus *Aloe* was due to a similar biogeographic history as the one inferred for *Aloe*, which underwent a series of radiations from a common ancestor in southern Africa from ∼15 Mya, before finally reaching the Arabian Peninsula ∼5 Mya (Grace et al., 2015).

*Aloidendron* species occur in either arid or humid thicket/forest habitats. Three closely related southern African desert species, *A. dichotomum* (Masson) Klopper & Gideon F.Sm., *Aloidendron ramosissimum* (Pillans) Klopper & Gideon F.Sm. and *Aloidendron pillansii* (L. Guthrie) Klopper & Gideon F.Sm., occur on rocky slopes in the arid Northern Cape province of South Africa and in southern Namibia to approximately 1,500 m (van Wyk and Smith, 2001). A fourth arid species, *A. sabaeum*, is widespread along the escarpment of the Arabian Peninsula, extending from the Aseer Province in southern Saudi Arabia to Hardramaut in eastern Yemen, and occurs at a range of altitudes from 150 to 2,000 m (Reynolds, 1966; Carter et al., 2011). In southern Africa, the thicket- and forest-dwelling species occur on sandy soils at sea level or low altitudes in the Eastern Cape and KwaZulu-Natal provinces of eastern South Africa, extending northwards into Eswatini and Mozambique. These include *Aloidendron barberae* (Dyer) Klopper & Gideon F.Sm. (coastal thicket, grassland) and *Aloidendron tongaensis* (van Jaarsv.) Klopper & Gideon F.Sm. (sand forest). A third forest species, *A. eminens*, is known only from a narrow distribution in Erigavo District, Somalia, where it occurs on forested slopes at altitudes of 1,550–1,830 m (Carter et al., 2011) (**Figure 1**).

The evolutionary relationships underlying the fragmented distribution of arborescent aloes have hitherto remained unconsidered in a phylogenetic context. One of the reasons for this knowledge gap is that long-lived succulent phanerophytes such as *Aloidendron* may reach flowering maturity only after a decade or more and/or rarely flower in cultivation, posing a challenge to scholars who are reliant on specimens verified with flowering material for research. We used a museomics approach to overcome this challenge. Museum collections globally have doubled since 1960 (Goodwin et al., 2015) and represent a rich source of plant material for research. Advances in -omics technologies now allow the exploitation of old, poorly preserved and fragmentary plant material to be included in molecular studies of evolution, systematics and taxonomy. Whole chloroplast genomes of preserved herbarium specimens and living plants were sequenced to illuminate phylogenetic relationships among tree aloes. The inclusion of type specimens and verified living collections of *Aloidendron* and *Aloe* allowed us to produce a phylogenomic hypothesis with which we could assess the taxonomic placement of all seven *Aloidendron* species and *A. suzannae*.

## Methods

### Sampling, DNA Extraction and Library Preparation

Plant material for investigation was obtained from herbarium specimens at the Herbarium at Kew (K) and accessions of known provenance grown in curated living collections (**Table 1**). Vouchers of *ex hort* material were deposited at the Herbarium at Kew (K); the H.G.W.J. Schweickerdt Herbarium at the University of Pretoria (PRU), South Africa; and the herbarium of the Sukkulenten-Sammlung in Zurich, Switzerland (**Table 1**). The sampling included 26 species in total: six out of the seven species of *Aloidendron*, 10 species of *Aloe* representing the major clades and geographical distribution, the monotypic *A. suzannae* and *Bulbine frutescens* (L.) Willd. as the outgroup for the phylogeny.

**Table 1:** Accession data of samples used in the museomic study of *Aloidendron* and related genera.

Genus	Herbarium (H) or living (L) material	Living collection and accession number^1^	Collector’s number	Distribution
*Aloe acutissima* H. Perrier	L	2010-1941, Kew	Grace 252	Madagascar
*Aloe arborescens* Mill.	L	P2012-5002, Copenhagen	Grace 195	South Africa, Lesotho
*Aloe broomii* Schönland	L	P2012-5005, Copenhagen	Grace 196	South Africa
*Aloe camperi* Schweinfurth.	L	1973-13456, Kew	Grace 254	Eritrea, Ethiopia
*Aloe castellorum* J.R.I. Wood	L	1981-2893, Kew	Grace 255	Saudi Arabia, Yemen
*Aloe elegans* Tod.	L	1984-171, Kew	Grace 258	Eritrea, Ethiopia
*Aloe gariepensis* Pillans	L	–	Grace 275	Namibia, South Africa
*Aloe greatheadii* Schönland	L	–	Grace 58	South Africa, Eswatini, Zimbabwe, Mozambique, Malawi, Botswana, Democratic Republic of Congo
*Aloe officinalis* Forssk.	L	1975-4505, Kew	Grace 262	Saudi Arabia, Yemen
*Aloe thraskii* Baker	L	P2012-5030, Copenhagen	Grace 216	South Africa
*Aloestrela suzannae* (Decary) Molteno & Gideon F.Sm	L	2012-675, Kew	Grace 272	Madagascar
*A. suzannae* (Decary) Molteno & Gideon F.Sm	H	–	Decary 2913 (type)	Madagascar
*Aloidendron barberae* (Dyer) Klopper & Gideon F.Sm.	H	–	Unknown (type)	South Africa, Mozambique, Eswatini
*A. barberae* (Dyer) Klopper & Gideon F.Sm.	L	1947-29913, Kew	Grace 292	South Africa, Mozambique, Eswatini
*A. barberae* (Dyer) Klopper & Gideon F.Sm.	L	1947-29913, Kew	Grace 292	South Africa, Mozambique, Eswatini
*Aloidendron dichotomum* (Masson) Klopper & Gideon F.Sm.	L	2015-832, Kew	Grace 289	Namibia, South Africa
*A. dichotomum* (Masson) Klopper & Gideon F.Sm.	H	–	Reynolds 5400	Namibia, South Africa
*Aloidendron eminens* (Reynolds & P.R.O. Bally) Klopper & Gideon F.Sm.	L	1981-895, Kew	Grace 296	Somalia
*A. eminens* (Reynolds & P.R.O. Bally) Klopper & Gideon F.Sm.	H	–	Reynolds 8435 (type)	Somalia
*Aloidendron pillansii* (L. Guthrie) Klopper & Gideon F.Sm.	L	SSZ 993355/6, SSZ	–	Namibia, South Africa
*A. pillansii* (L. Guthrie) Klopper & Gideon F.Sm.	H	–	Pillans 5012 (type)	Namibia, South Africa
*Aloidendron ramosissimum* (Pillans) Klopper & Gideon F.Sm.	H	–	Reynolds 2547 (type)	Namibia, South Africa
*Aloidendron sabaeum* (Schweinf.) Boatwr. & J.C. Manning	L	–	Van Wyk 14198	Saudi Arabia, Yemen
*A. sabaeum* (Schweinf.) Boatwr. & J.C. Manning	H	–	Schweinfurthii 941 (type)	Saudi Arabia, Yemen
*A. sabaeum* (Schweinf.) Boatwr. & J.C. Manning	L	1973-2559, Kew	Grace 299	Saudi Arabia, Yemen
*Bulbine frutescens* (L.) Willd.	L	1973-3211, Kew	Grace 260	South Africa

^1^Copenhagen, Copenhagen Botanic Garden, Denmark; Kew, Royal Botanic Gardens, Kew, United Kingdom; SSZ, Sukkulenten-Sammlung Zurich, Switzerland.

To extract total genomic DNA from living plants, ∼0.2 g of silica-dried leaf tissue was powdered with metallic beads in a Qiagen TissueLyser (Hilden, Germany). DNA was then extracted and purified with the Qiagen DNeasy plant DNA kit (Manchester, UK) according to the manufacturer’s protocol. We used a different method for old herbarium specimens, which are expected to contain highly degraded DNA. First, ∼0.1 g of herbarium material was finely ground in a mortar, using liquid nitrogen. Genomic DNA was then extracted from the resulting powder using a modified 2× CTAB method (Doyle and Doyle, 1987) where the DNA was precipitated with isopropanol for 2 weeks at −80°C. The extracted DNA was cleaned with AMPure XP Beckman Coulter beads, using a 1:1 beads-to-DNA ratio in order to maximise the retrieval of small DNA fragments. The DNA concentration was quantified using the QuantiFluor dsDNA System (Promega) on a Quantus fluorometer (Invitrogen, UK), and fragment size was assessed by electrophoresis in a 1% agarose gel.

A genome skimming approach was used, in which genomic DNA is sequenced sufficiently to retrieve genomic regions present in high copy numbers, notably the plastid genome (Twyford and Ness, 2017; Weitemier et al., 2014). The DNA extracted from the living material was sheared in a Covaris Focused-ultrasonicator M220 into Covaris microTUBE AFA Fiber Pre-Slit Snap-Cap tubes with the following parameters: peak power 50.0, duty factor 20, cycles/burst 200 and treatment time 60 s, to achieve an average DNA fragment length of 500 bp. In contrast, the DNA from the herbarium material was already degraded (<300 bp) and therefore not sheared. The paired-end DNA libraries were prepared using the NEBNext Ultra II Library prep kit for Illumina (New England Biolabs, MA) with insert sizes of ∼200 bp for herbarium samples (highly fragmented and degraded DNA) and ∼500 bp for fresh silica-dried samples (high molecular weight and intact DNA). Importantly, a 10-fold adaptor dilution was used for the herbarium samples to avoid the formation of adaptor dimers during the library preparation. Library quality (concentration and insert size) was assessed with a Quantus fluorometer (Invitrogen, UK) and an Agilent 4200 TapeStation (Stockport, UK). Sequencing was performed on an Illumina MiSeq platform (Illumina, UK) to generate at least two million 150-bp-long paired-end reads for the herbarium samples and at least 5.7 million 250-bp-long paired-end reads for the silica-dried samples.

### Data Cleaning, Plastome Assembly and Annotation

Adaptors were removed and low-quality sequences trimmed using Trimmomatic 0.310 with the parameters MAXINFO:40:0.85 HEADCROP:1 MINLEN:36. The reads of the *A. pillansii* AT12 sample were assembled using the Fast-Plast (https://github.com/mrmckain/Fast-Plast) (McKain and Wilson, 2019) pipeline, which yielded one plastome contig. The circularity and integrity of the contig were checked by mapping the reads back to it using Geneious R8 (https://www.geneious.com). The plastome annotation was performed with GeSeq (Tillich et al., 2017), using the published plastome of *Agave attenuata* (NC_032696.1) (McKain et al., 2016) as a reference. The annotations were then inspected in Geneious R8 (https://www.geneious.com) and edited manually if necessary. The annotated plastid genome sequence of *A. pillansii* was deposited in GenBank (MN276325). The plastome map was drawn with OGDraw v.1.2 (Lohse et al., 2007).

### Phylogenetic Analysis

The annotated plastome of *A. pillansii* was split into 91 coding (exons) and 113 non-coding (introns and intergenic spacers) regions, which were combined in a coding reference file and a non-coding reference file, respectively. The nuclear ribosomal ITS1_58S_ITS2 region of *A. pillansii* was added to the non-coding reference file. Each reference file was then used to recover homologous regions in all other samples (**Table 1**) using the pipeline HybPiper v.1.2 (Johnson et al., 2016). The pipeline mapped the clean reads of each sample against each reference locus using BWA (Li and Durbin, 2009), separately assembled the reads corresponding to each locus using SPAdes (v.3.11.1) (Bankevich et al., 2012), and, for each locus, combined the homologous regions found in all samples in a locus matrix. All 205 plastid and nuclear locus matrices were individually aligned using MAFFT 7 (Katoh and Standley, 2013) and inspected in Geneious R8 (**Supplementary File S1**). The internal transcribed spacer (ITS) matrix was analysed separately. Plastid coding and non-coding loci were concatenated into one plastid matrix of 148,678 nucleotide sites. A second plastid matrix was then obtained by removing from the first matrix all nucleotide sites with more than 75% gaps and five taxa that had a high proportion of missing data (*Aloe arborescens* A8, *Aloe broomii* A13, *Aloe thraskii* A83, *A. barberae* A10 and *A. sabaeum* AT3; see **Table 1** and **Results**). The ITS alignment, consisting of 733 nucleotide sites, was analysed separately to allow the detection of nucleocytoplasmic conflicts.

The concatenated plastid matrix and the ITS matrix were each submitted to maximum-likelihood phylogenetic analysis using RAxML 8.2.9 (Stamatakis, 2014) *via* the CIPRES Science Gateway (Miller et al., 2010), with a GTRGAMMA evolutionary model specified. The plastid matrix was analysed as two partitions corresponding to the coding and non-coding regions. Node supports were estimated using a rapid bootstrap analysis with 1,000 replicates. All trees were rooted using *B. frutescens* as the outgroup, using the tree rooting function pxrr implemented in the phyx tool (Brown et al., 2017).

## Results

The museomics approach sampling preserved herbarium samples and living plants in curated botanical collections yielded 205 loci for 26 samples representing six species of *Aloidendron*, 10 of *Aloe*, *A*. *suzannae* and the outgroup *B. frutescens* (**Table 1**). We sequenced the type specimens of five out of seven species of *Aloidendron*, the exceptions being *A. dichotomum* and *A. tongaensis*. However, we did include a specimen of *A. dichotomum* collected in 1949 by G.W. Reynolds, which he described as “typical” (Reynolds, 1950: 490). The oldest among the samples used in this study was a herbarium specimen prepared in approximately 1874 at the Royal Botanic Gardens, Kew, from a cutting of *A. barberae*, sent from Kwa-Zulu-Natal in South Africa. Another historic sample was the type specimen of *A. sabaeum*, collected in February 1889 by George Schweinfurth. Other herbarium specimens sampled were all collected in the 20th century (**Table 1**). Despite optimisations, a modern sample of *A. tongaensis* (Van Jaarsveld, 2010), a species described in 2010, could not be successfully sequenced.

**Figure 2:**
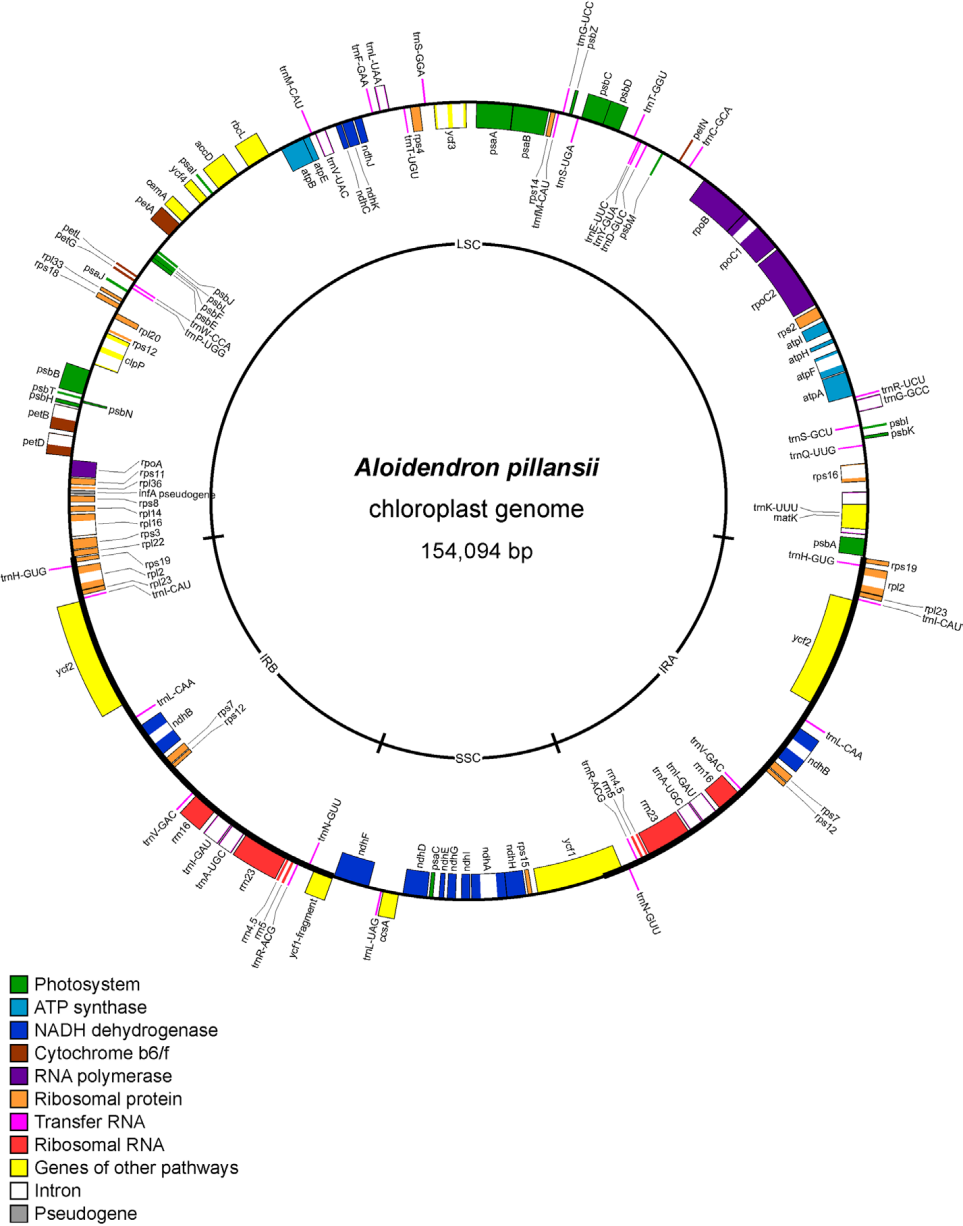
Gene map of *Aloidendron pillansii* chloroplast genome (GenBank MN276325). Genes illustrated inside the circle are transcribed in a clockwise direction relative to the figure, and genes outside the circle are transcribed in an anticlockwise direction.

The assembled plastome of *A. pillansii* was 154,094 bp long with a large single-copy (LSC) region of 84,002 bp and a short single-copy (SSC) region of 16,952 bp separated by two inverted repeats (IRs) of 26,570 bp (**Figure 2**). The LSC-IR_B_ boundary is located between the rpl22 and rpl19 ribosomal protein genes, and the LSC-IR_A_ boundary between rpl19 and psbA, while the SSC-IR_A_ and SSC-IR_B_ boundaries are within the *ycf1* gene.

**Figure 3:**
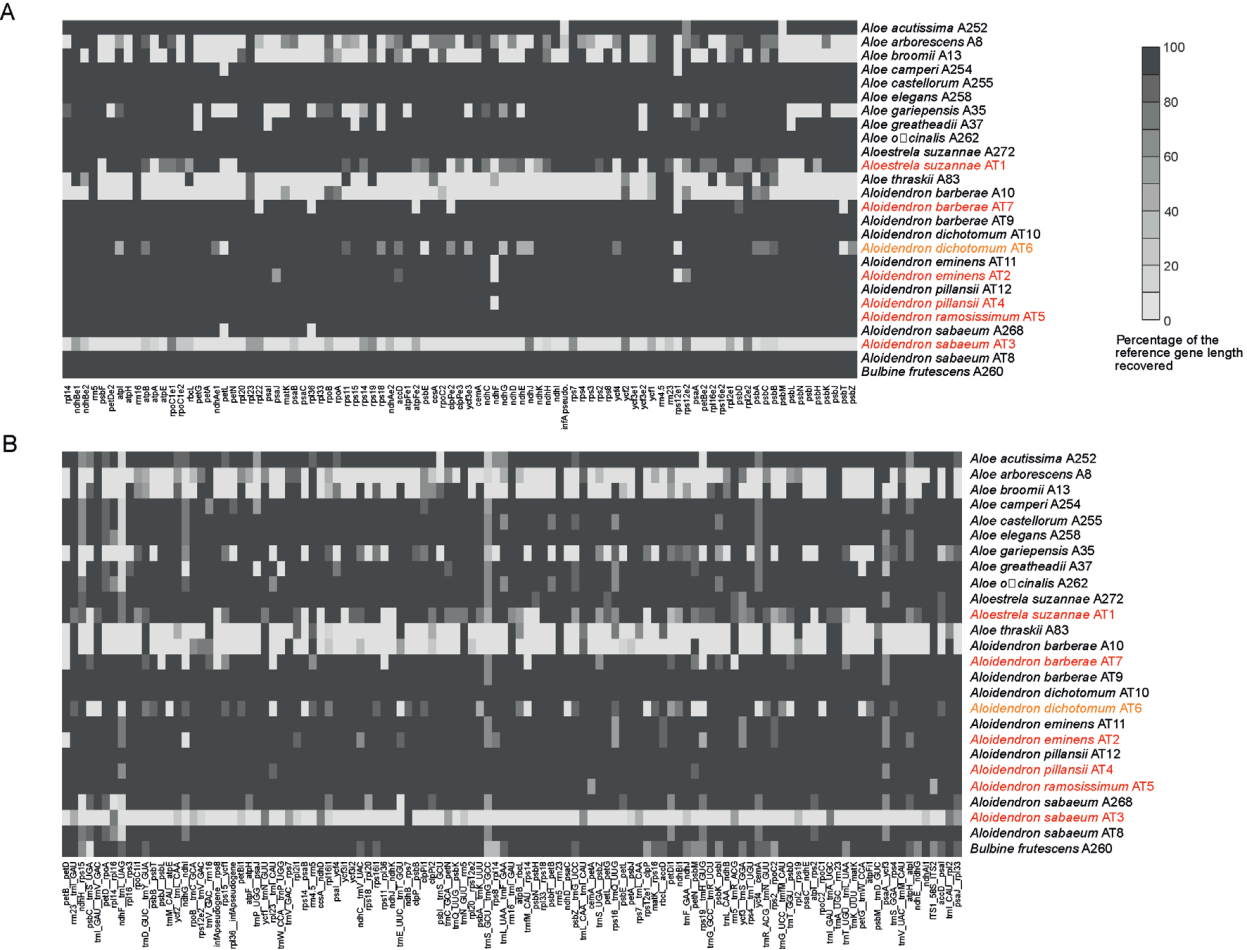
Heatmaps showing gene recovery efficiency for **(A)** coding plastid loci and **(B)** non-coding plastid loci and the nuclear ribosomal internal transcribed spacer (ITS) region in 25 species of Asphodelaceae. Columns represent genes, and each row is one sample. Shading indicates the percentage of the reference locus length coverage. Samples retrieved from type or historical specimens are highlighted in red and orange, respectively.

Using the genes and intergenic regions of the plastome of *A. pillansii* as a reference allowed us to recover their homologous regions in most of the other samples (**Figure 3**). For each sample, the length of each recovered region was compared to the length of that region in *A. pillansii* (**Table 2**). For 17 samples, including the types of *A. barberae*, *A. eminens*, *A. pillansii* and *A. ramosissimum*, we could retrieve 181 to 203 of the 203 plastid loci at >80% of their length (**Figure 3**). Three samples, including the type of *A. suzannae* and the historical specimen of *A. dichotomum*, had an intermediate recovery (127 to 160 loci), while the type of *A. sabaeum* and four other samples had only 1 to 68 plastid loci recovered at >80% (**Table 2**). When considering loci spanning >50% of the reference length, between 0 and 36 additional loci could be recovered, and the sample performance ranking was identical, with the same 17 samples performing very well (188–203 loci), the same three intermediate samples (143–177 loci), and the same five samples with low recovery (22–81 loci; **Table 2**). The recovery of the non-coding regions was somewhat less successful than that of the coding regions (**Figure 3**), with the percentage of loci recovered at >80% of the reference length being almost always lower for non-coding loci than for coding loci, by up to 13 points in *A. arborescens* (**Table 2**). A notable exception was the low-performing *A. barberae* sample A10, for which 15.2% of the non-coding loci were recovered, against only 8% of the coding loci (**Table 2**). The ITS region could be recovered in all samples at >98.5% of the reference length, except for the type of *A. sabaeum*, where it was not recovered, and the type of *A. ramosissimum* and the outgroup *Bulbine*, where only 41.4% and 64.3% of the reference region could be found, respectively (**Figure 3**; **Table 2**).

**Table 2:** Locus recovery in *Aloidendron* and related genera compared to the number and length of the reference loci. Samples from type and historic specimens are shown in red and orange, respectively and the reference in bold.

Sample	Total number of plastid loci recovered at >50%	Total number of plastid loci recovered at >80%	% Plastid coding loci recovered at 80%	% Plastid non-coding loci recovered at 80%	% Reference ITS length recovered
*Aloe acutissima* A252	197	189	96.7	90.2	100
*Aloe arborescens* A8	77	45	29.7	16.1	100
*Aloe broomii* A13	81	68	34.1	33.0	100
*Aloe camperi* A254	200	192	97.8	92.0	98.5
*Aloe castellorum* A255	202	194	100.0	92.0	98.5
*Aloe elegans* A258	202	195	100.0	92.9	98.5
*Aloe gariepensis* A35	143	127	69.2	57.1	98.5
*Aloe greatheadii* A37	190	186	93.4	90.2	98.5
*Aloe officinalis* A262	202	193	100.0	91.1	98.5
*Aloe thraskii* A83	41	31	15.4	15.2	98.5
*Aloestrela suzannae* A272	203	199	100.0	96.4	100
*A. suzannae* AT1	170	134	65.9	66.1	100
*Aloidendron barberae* A10	35	25	8.8	15.2	100
*A. barberae* AT7	188	181	93.4	85.7	100
*A. barberae* AT9	203	201	100.0	98.2	100
*Aloidendron dichotomum* AT10	203	203	100.0	100.0	100
*A. dichotomum* AT6	177	160	80.2	77.7	100
*Aloidendron eminens* AT11	202	197	98.9	95.5	99.9
*A. eminens* AT2	198	188	95.6	90.2	100
***Aloidendron pillansii* AT12**	203	203	100.0	100.0	100
*A. pillansii* AT4	202	200	98.9	98.2	100
*Aloidendron ramosissimum* AT5	203	202	100.0	99.1	41.4
*Aloidendron sabaeum* A268	197	188	97.8	88.4	100
*A. sabaeum* AT3	22	1	0.0	0.9	0
*A. sabaeum* AT8	202	194	100.0	92.0	100
*Bulbine frutescens* A260	200	190	100.0	88.4	64.3

**Figure 4:**
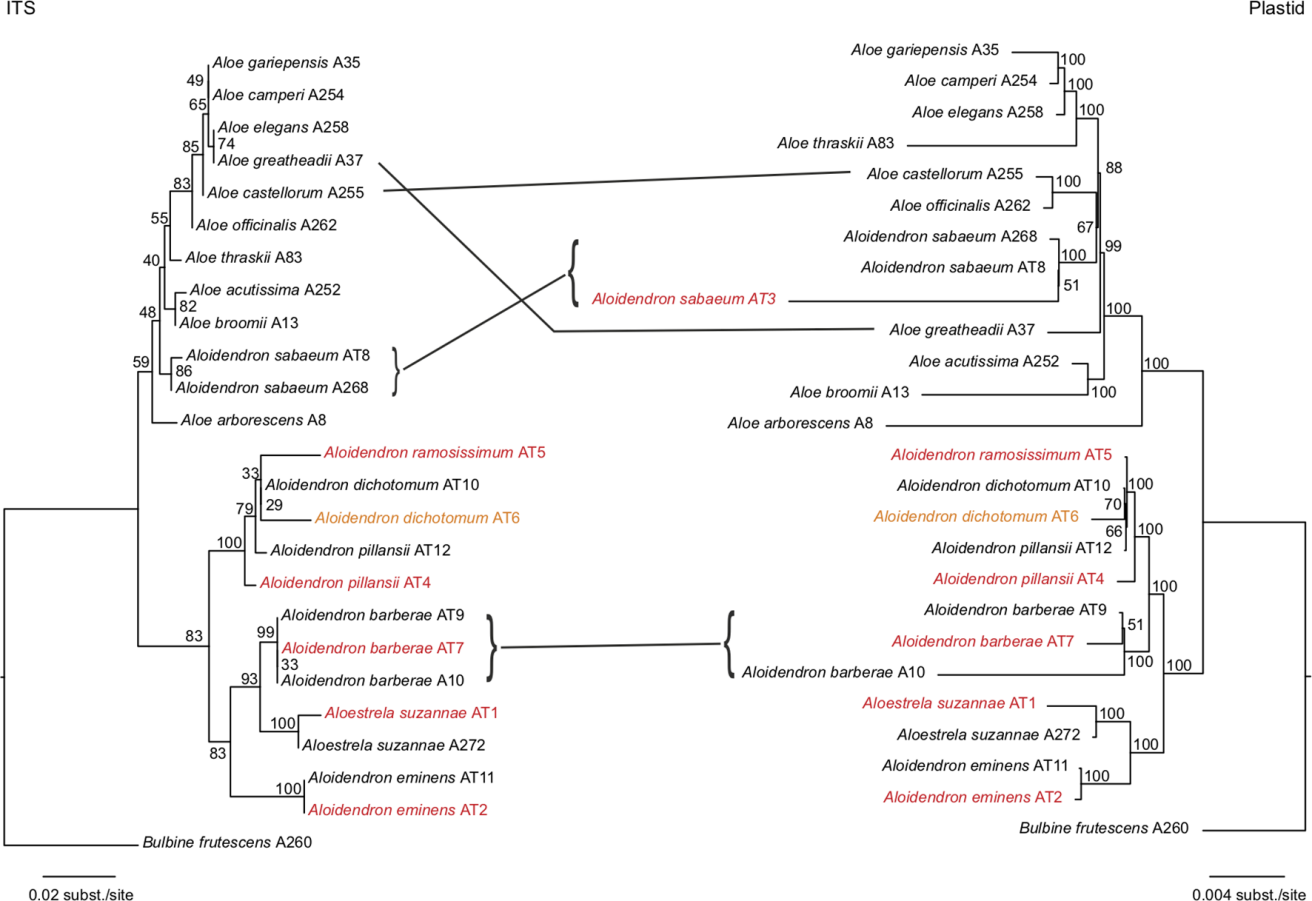
Maximum-likelihood trees estimated from 204 plastid loci and internal transcribed spacer (ITS), recovered for 25 samples representing *Aloidendron*, *Aloestrela* and *Aloe* with node supports expressed as bootstrap percentages. Samples retrieved from type or historical specimens are highlighted in red and orange, respectively.

**Figure 5:**
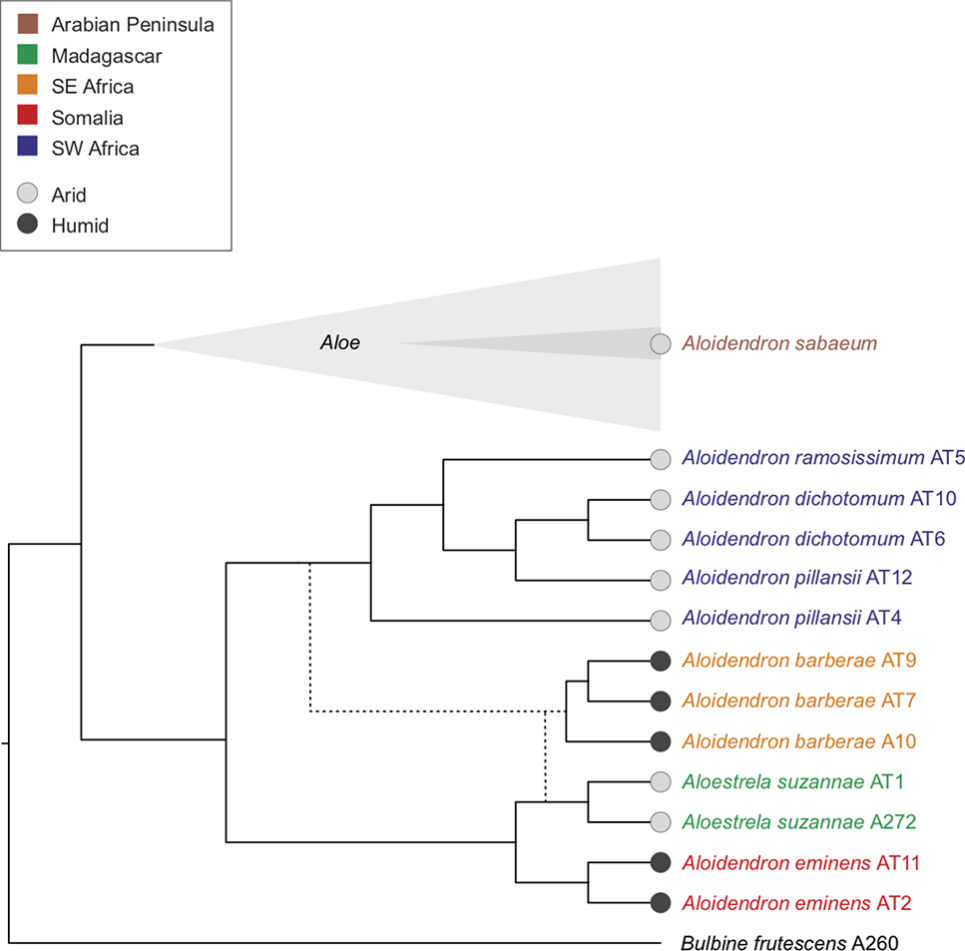
Summary tree topology of phylogenetic relationships among *Aloidendron* species, estimated from plastid loci and internal transcribed spacer (ITS) maximum-likelihood trees, showing geographical distribution and habitat preferences.

Maximum-likelihood trees were estimated from all plastid loci and ITS (**Figure 4**) and with gappy sites and sequences removed (**Figure S1**). The historical specimens and types always grouped with conspecific samples from fresh material (**Figure 4**), and long branches, possibly due to missing data (Darriba et al., 2016), did not affect the topology (**Figure S1**). A consensus of the plastid and ITS topologies focusing on the tree aloes with the geographical and ecological distribution of each species is shown in **Figure 5**. Both plastid and nuclear regions recovered two major well-supported clades, one consisting mainly of *Aloes* and the other comprising mostly *Aloidendron* species. However, neither of these genera was monophyletic (**Figure 4**), since the *Aloe* clade included *A. sabaeum* with BP = 59 (ITS) to BP = 100 (plastid) and the *Aloidendron* clade included *A. suzannae* with BP = 83 (ITS) to BP = 100 (plastid).

The focus of this study, *Aloidendron*, consisted of two sub-clades recovered in both the ITS and the plastid phylogenies: a southwestern African desert clade (BP = 100) comprising the morphologically similar and sympatric desert species *A. dichotomum*, *A. ramosissimum* and *A. pillansii* and an eastern clade (BP = 83 or 100) comprising the narrowly endemic Somalian humid thicket species *A. eminens* and the Madagascar endemic arid thicket species *A. suzannae*. The southern African humid forest/thicket species *A. barberae* was recovered in the desert clade in the plastid tree (BP = 100), but in the eastern clade in the ITS tree, sister to *A. suzannae* (BP = 93; **Figure 4**). Among the species of the desert clade, the two samples of *A. pillansii* were not monophyletic, and although sample AT4 was recovered as sister to the other species in both trees, sample AT12 was either sister to *A. ramosissimum* + *A. dichotomum* in the ITS tree (BP = 33) or sister to *A. dichotomum* in the plastid tree (BP = 66). The relationships in the *Aloe* clade were similar in the ITS and plastid tree, except for three well-supported (BP ≥ 70) nucleocytoplasmic conflicts (**Figure 4**). *Aloe officinalis* and *Aloe castellorum* were recovered as sister species in the plastid tree, but in the ITS tree, they formed a grade around the clade comprising *Aloe gariepensis*, *Aloe camperi*, *Aloe elegans* and *Aloe greatheadii*. The latter species was not part of this clade in the plastid tree but instead was inferred to have diverged earlier. Finally, although *A. sabaeum* was nested in *Aloe* in both trees, its precise position varied from being sister to all sampled *Aloes* except *A. arborescens* in the ITS tree (BP = 48) and being sister to *A. officinalis* + *A. castellorum* in the plastid tree (BP = 67).

## Discussion

Museomic, or herbariomic, approaches based on preserved botanical collections can fill critical gaps in phylogenetic sampling, particularly when species are extinct in the wild (Silva et al., 2017) or taxonomically complex (Chomicki and Renner, 2014). In this study, sequencing historic reference material, including the type specimens of five *Aloidendron* species, minimised risks of misidentification which may otherwise apply to slow-growing plants in cultivation, particularly when they flower infrequently. All species represented by both herbarium specimens and living plants were consistently recovered together in phylogenetic trees (**Figures 4**, **5**), highlighting, in turn, the value of well-curated living collections in plant systematics. In recent years, the value of degraded and/or historic botanical specimens has been illustrated primarily in the context of crop domestication (e.g. the potato, *Solanum tuberosum*) (Gutaker et al., 2019). Although DNA extraction from herbarium specimens and the fragmentary nature of their DNA pose persistent challenges to sequencing, the sensitivity of high-throughput sequencing technologies now makes it possible to include herbarium samples in molecular systematic studies. Our herbariomic approach allowed for the recovery of many coding and non-coding plastid loci as well as the nuclear ITS region from most samples, including the historical ones. In addition, we showed that genome skimming can be successfully used to sequence plastid genomes in Asphodelaceae. The *A. pillansii* plastome assembled for the study complies with the typical angiosperm chloroplast arrangement which is characterised by large and small single-copy regions separated by two IR regions (IR_A_ and IR_B_) (Palmer, 1985). The loss of rpl32 from members of Asphodelaceae (Steele et al., 2012) was confirmed in this species (**Figure 2**).

The plastid and nuclear phylogenies inferred from our herbariomic data provide compelling insights into the taxonomy and speciation of tree aloes (**Figure 4**). Arborescent aloes conforming to Raunkier’s succulent phanerophyte life form (Raunkier, 1934) were recently elevated to the genus rank in *Aloidendron* and *Kumara* (Grace et al., 2013). *Kumara* is a distinctive monophyletic Cape genus comprising the arborescent *K. plicatilis* and the short-stemmed shrub, *Kumara haemanthifolia* (Marloth & A. Berger) Boatwr. & J.C. Manning (Manning et al., 2014). Previously, all seven *Aloidendron* species were included in *Aloe* sections Dracaloe and Aloidendron, as circumscribed by Alwyn Berger (Berger, 1908) and Gilbert Reynolds (Reynolds, 1950). However, the phylogenetic placements that we recovered for *A. sabaeum* and *A. suzannae* question these two taxonomic treatments of tree aloes (**Figure 4**).

The phylogenetic placement of the Arabian species *A. sabaeum* in *Aloe* has nomenclatural implications (Smith et al., 2019). *Aloe sabaea* Schweinf. was transferred to *Aloidendron* (Manning et al., 2014) following Gilbert Reynolds’ grouping (Reynolds, 1966) grouping of this species with the eastern tree aloes [*A. eminens* and *Aloe bainesii* (= *A. barberae*)] on the basis of an erroneous record of its height of up to 9 m, whereas in fact *A. sabaea* reaches only 3 m on maturity (Carter et al., 2011). The concept of *Aloidendron sensu* Grace et al. (2013) excluded *A. sabaea* on this basis and is supported by our study. In addition, the recognition of the Madagascar endemic *A. suzannae* (= *Aloe suzannae* L.) as a monotypic genus (Smith and Molteno, 2019) was not supported in this first effort to sequence it, given that it was recovered as sister to *A. eminens* within the *Aloidendron* clade. Finally, species boundaries in *Aloidendron* may also need to be reassessed, as suggested by the non-monophyly of the two accessions of *A. pillansii* included in our study. A more detailed analysis of phylogenomic and morphological trait data may help to resolve the taxonomic rank of potentially conspecific taxa in the southwestern desert clade. Hybridisation events or conflicting gene histories may require closer consideration. For instance, the conflicting positions of *A. barberae* and of some *Aloe* species between the nuclear and plastid trees (**Figure 4**) may be indicative of hybridisation followed by chloroplast capture or of incomplete lineage sorting. Expanded sampling and sequencing of additional nuclear genes could help in tackling these challenges. Our demonstration of high-throughput sequencing applied to Asphodelaceae herbarium material opens opportunities for a phylogenomic study of the family to underpin the necessary taxonomic revision.

Our phylogeny also provides a new context in which to assess the biogeography of tree aloes and the evolution of their arborescent habit and ecological preferences (**Figure 5**). The four southwestern African desert *Aloidendron* species share short, ventricose flowers and are pollinated by short-billed birds, whereas the eastern humid forest species *A. tongaensis* and *A. eminens* have longer, curved flowers in pendant racemes and are pollinated by sunbirds (Botes et al., 2008; Van Jaarsveld and Judd, 2015). This led to an interpretation of the desert and humid forest groups as two distinct evolutionary lineages (Van Jaarsveld and Judd, 2015), which was not, however, supported by our study. Instead, the two clades in *Aloidendron* correspond to the two geographical regions in which the genus is represented: southern Africa and east Africa/Madagascar. The geographical trend observed in our phylogeny of *Aloidendron* has also been observed in the related genus *Aloe* (Grace et al., 2015).

The range disjunction of *Aloidendron* between southern Africa and the Horn of Africa (**Figures 1** and **5**) conforms to the Rand Flora pattern, in which related taxa that were once linked by an arid corridor between southern Africa and north-east Africa are now widely separated (Sanmartin et al., 2010; Pokorny et al., 2015). Intense aridification during the Miocene and Pliocene was the major driver of the disjunct distributions shared by 12 angiosperm families in the African flora (Sanmartin et al., 2010; Pokorny et al., 2015), and these conditions have been linked to successive radiations of *Aloe* in Africa (Grace et al., 2015). One hypothesis to explain the geographical pattern found in this study, therefore, is that extant *Aloidendron* are the relics of a previously widespread group of arborescent aloes that were linked by the arid corridor reaching from south-west Africa to the eastern seaboard of Africa. However, a more plausible scenario is that *Aloidendron* followed the same pattern as *Aloe* (Grace et al., 2015), with early-diverging tree aloes undergoing at least one dispersal event from southern Africa northwards to reach the Horn of Africa and Madagascar, at the distal regions of the range for tree aloes. It is also possible that the extant diversity of *Aloidendron* in southern Africa reflects both extinctions in the desert regions and diversification in the East African range. The phylogenetic proximity of *A. suzannae* to *A. eminens* is therefore explained by a common ancestor on the African continent, from ancient radiations of the arborescent habit from a southern African origin. *A. sabaeum* (= *Aloe sabaea*), in contrast, likely evolved from the radiation of *Aloe* on the Arabian Peninsula within the last 5 million years (Grace et al., 2015) (**Figures 4** and **5**). The water-storing leaves of arborescent succulent phanerophytes may have been an important adaptation that allowed aloes to migrate northwards during Miocene drought conditions from southern Africa to the Horn of Africa, eastern Indian Ocean Islands and, finally, the Arabian Peninsula (Grace et al., 2015; Dee et al., 2018). Further biogeographic studies of the aloes of Madagascar and other Indian Ocean islands will likely determine whether the evolutionary history of *Aloe* and related genera is similar to that of continental Africa (Grace et al., 2015;Dee et al., 2018), with earlier radiations of arborescent lineages followed by at least one rapid radiation of short-stemmed chamerophyte taxa.

Our findings emphasise the value of herbarium specimens in monographing a taxonomically complex, slow-growing, and diverse succulent group. Further work to expand the phylogenetic sampling of a wider variety of life forms among Asphodelaceae subf. Alooideae will add insights into their evolutionary history.

## Data Availability Statement

The *Aloidendron pillansii* chloroplast, complete genome has been deposited in the GenBank under accession number MN276325.

## Author Contributions

OG designed the study. PM, RD and OG conducted experimental work. SB, PM and OG analyzed and interpreted the results. OG, PM and SB prepared the manuscript which was agreed on by all authors.

## Funding

This study was supported by a grant to OG from the Systematics Association Research Fund.

## Conflict of Interest

The authors declare that the research was conducted in the absence of any commercial or financial relationships that could be construed as a potential conflict of interest.

## Supplementary Material

Supplementary File S1 Alignments of ITS and plastid sequence data of *Aloidendron* and related genera analysed in this study.

Supplementary Figure S1 Maximum likelihood tree estimated from plastid loci with <75% gaps recovered for 20 samples representing 14 species of *Aloidendron*, *Aloestrela* and *Aloe* with node supports expressed as bootstrap percentages.
