# Supplementary figures and images for: Museomics Clarifies the Classification of Aloidendron (Asphodelaceae), the Iconic African Tree Aloes
Source: Front Plant Sci. 2019 Oct 15;10:1227. doi: 10.3389/fpls.2019.01227 (PMC6803536; doi:10.3389/fpls.2019.01227)

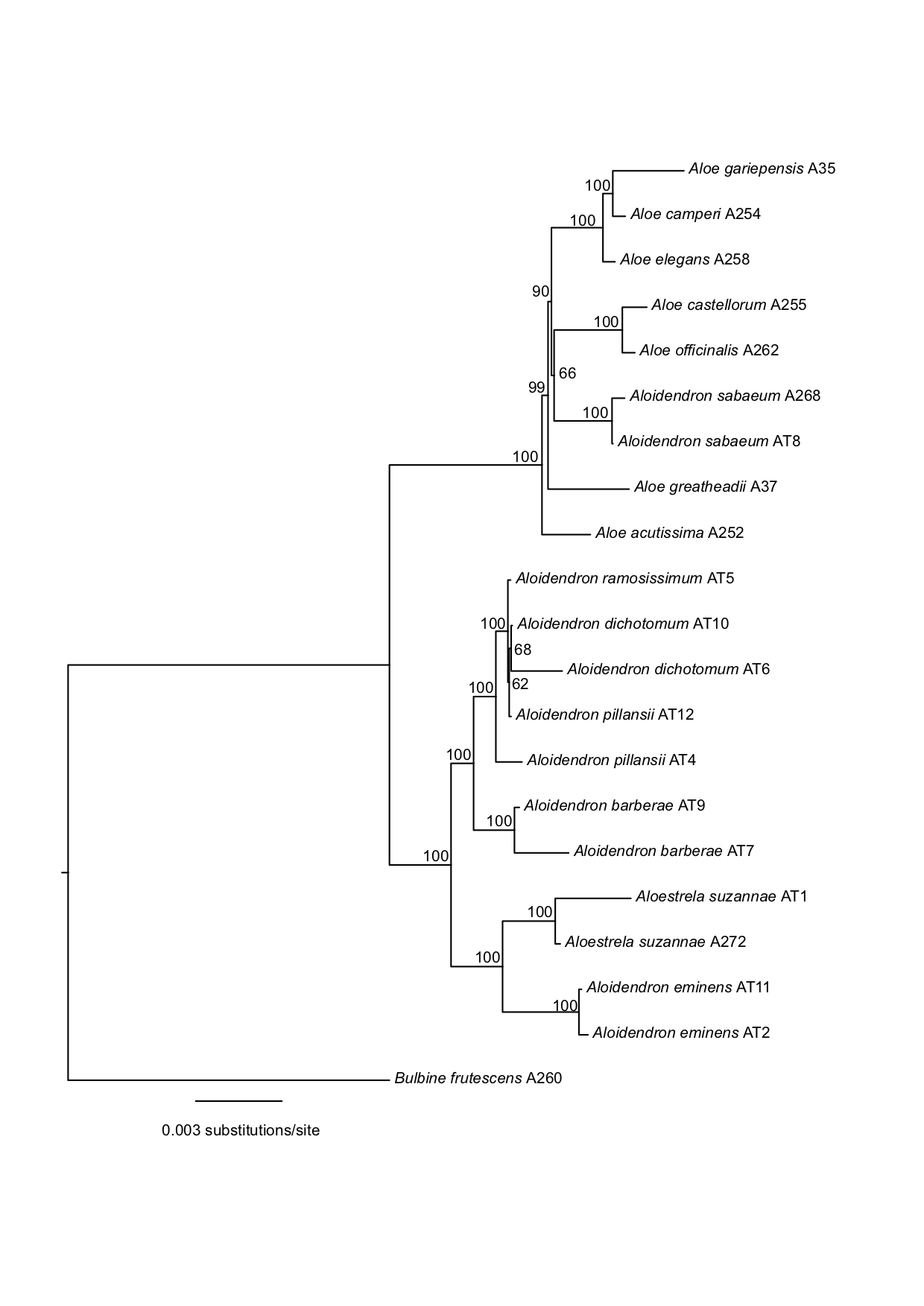

Supplement: Supplementary Figure S1 — Maximum likelihood tree estimated from plastid loci with <75% gaps recovered for 20 samples representing 14 species of Aloidendron, Aloestrela and Aloe with node supports expressed as bootstrap percentages. [file Image_1.tif]
